# Complete mitochondrial genome analysis of *Aquilonastra batheri* (Echinodermata, Asteroidea, Valvatida)

**DOI:** 10.1080/23802359.2018.1507655

**Published:** 2018-10-29

**Authors:** Taekjun Lee, Sook Shin

**Affiliations:** a Marine Biological Resource Institute, Sahmyook University, Seoul, Korea;; b Division of Life Sciences, College of Life Sciences and Biotechnology, Korea University, Seoul, Korea;; c Department of Chemistry Life Science, Sahmyook University, Seoul, Korea

**Keywords:** Echinodermata, Asterinidae, Aquilonastra, sea star, complete mitogenome

## Abstract

In this study, we determined the complete mitochondrial genome sequences of *Aquilonastra batheri*. This is the first study on mitochondrial genome sequencing of the genus *Aquilonastra* belonging to the family Asterinidae, order Valvatida, and class Asteroidea. The complete mitogenome of *A. batheri* was 16,463 bp and consisted of 13 protein-coding genes (PCGs), 22 tRNAs, and two rRNAs. The orders of PCGs and rRNA genes were identical to those of nine recorded mitogenomes of asteroids. The phylogenetic tree determined by the maximum likelihood method revealed that *A. batheri* was clearly grouped with *Patiria pectinifera*, which belongs to the family Asterinidae.

A sea star genus *Aquilonastra* was removed from the genus *Asterina* in 2004, based on a molecular and morphological revision (O’Loughlin and Waters 2004). A total of 31 species of the genus *Asterina* have been reported to date worldwide, and these species have extensive distribution, from Korea to Australia and Maldives, Arabian Sea, Red Sea, Indonesia, and Papua New Guinea (Clark 1921; O’Loughlin and Rowe 2006; Shin 2010; O’Loughlin and Bribiesca-Contreras 2015, 2017). *Aquilonastra batheri* is a common species found in the shallow water of Jeju-do, whose morphological characteristics are as follows: small sized (>30 mm), oral side flattened, with various marked irregular patterns on the aboral side (Shin 2010).

Specimens were collected by SCUBA diving at a depth of 21 m from the Munseom Island, Jeju-do in Korea (33°13′38″N, 126°34′08″E) on 29 March 2018. Specimens were deposited in the Marine Echinoderm Resources Bank of Korea (Seoul, Korea). The method of mitochondrial DNA extraction and amplification, next-generation sequencing (NGS), and genomic library conformed with those in the study by Lee and Shin (2018). Phylogenetic analyses of the dataset were performed using the maximum likelihood (ML) method with PhyML 3.1 (Guindon et al. 2010). The best-fit substitution was estimated using jModelTest 2.1.1 (Guindon and Gascuel 2003; Darriba et al. 2012) for the nucleotide dataset of 13 protein-coding genes (PCGs). For ML analyses, PhyML were used with the GTR + G model of substitution for the nucleotide dataset. Bootstrap resampling was performed using the rapid option with 1,000 iterations.

The complete mitogenome of *A. batheri* (GenBank accession No. MH507076) was 16,463 bp and contained 13 PCGs, 22 tRNA genes, and two rRNA genes. The order and direction of the genes were identical to those of the other nine complete mitogenomes of asteroid species. Eleven PCGs contained an ATG initiation codon (methionine), with the exception of NADH4L (ATC, isoleucine) and NADH3 (ATT, isoleucine). The TAA codon was the termination codon of nine PCGs, with the exceptions of COX2, NADH2, NADH5 (TAG), and CytB (Phenylalanine (TTC)+T).

To examine the phylogenetic relationships, the ML method was used based on the nucleotide sequences of 13 PCGs obtained from ten asteroids, including *A. batheri* (Figure 1), with two ophiuroids, *Astrospartus mediterraneus* (NC_013878) and *Ophiura luetkenii* (NC_005930), which were used as outgroups. In the phylogenetic tree, the ten asteroid mitogenomes were clearly divided with two ophiuroids (Figure 1). *Aquilonastra batheri* formed a distinct monophyly with *Patiria pectinifera* (NC_001627), which belonged to the family Asterinidae. Furthermore, they distinctly formed a monophyletic clade with *Acanthaster brevispinus* (NC_007789) and *A. planci* (NC_007788), which belongs to the identical order Valvatida (Figure 1).

**Figure 1.**
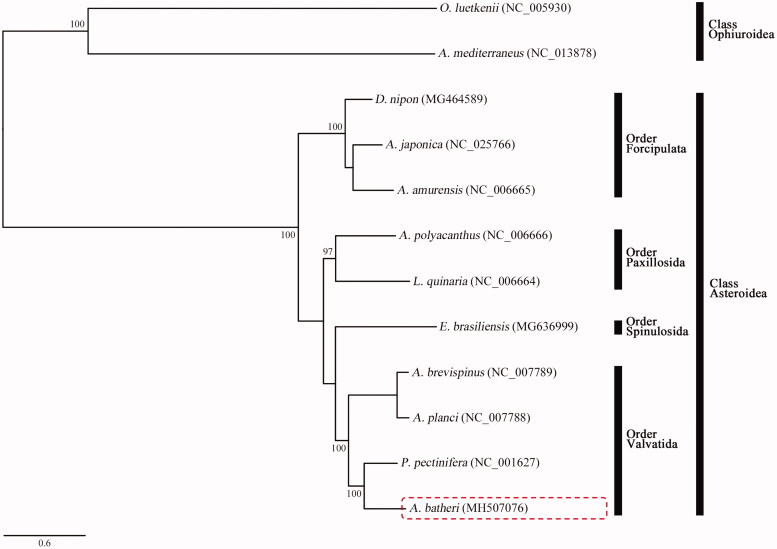
Phylogenetic tree constructed using the maximum likelihood (ML) method based on the nucleotide sequences of the complete mitogenomes of *Aquilonastra batheri* (MH507076) and eight other asteroids. The bootstrap values indicated on each node are >70.

## References

[CIT0001] Clark HL 1921 The echinoderm fauna of Torres Strait: its composition and its origin. Washington (DC): Publication of the Carnegie Institution of Washington.

[CIT0002] Darriba D, Taboada GL, Doallo R, Posada D 2012 jModelTest 2: more models, new heuristics and parallel computing. Nat Methods. 9:772 10.1038/nmeth.2109 PMC4594756 22847109

[CIT0003] Guindon S, Dufayard JF, Lefort V, Anisimova M, Hordijk W, Gascuel O 2010 New algorithms and methods to estimate maximum-likelihood phylogenies: assessing the performance of PhyML 3.0. System Biol. 59:307–321. 20525638 10.1093/sysbio/syq010

[CIT0004] Guindon S, Gascuel O 2003 A simple, fast, and accurate algorithm to estimate large phylogenies by maximum likelihood. System Biol. 52:696–704. 14530136 10.1080/10635150390235520

[CIT0005] Lee T, Shin S 2018 Complete mitochondrial genome analysis of *Distolasterias nipon* (Echinodermata, Asteroidea, Forcipulatida. Mitochondrial DNA. doi:10.1080/23802359.2018.1501313. PMC7800799 33474496

[CIT0006] O’Loughlin PM, Bribiesca-Contreras G 2015 New asterinid seastars from northwest Australia, with a revised key to *Aquilonastra* species (Echinodermata: Asteroidea). Memoirs of Museum Victoria. 73:27–40.

[CIT0007] O’Loughlin PM, Bribiesca-Contreras G 2017 New asterinid seastars from the western Pacific Ocean (Echinodermata: Asteroidea). Memoirs of Museum Victoria. 76:121–132.

[CIT0008] O’Loughlin PM, Waters JM 2004 A molecular and morphological revision of genera of Asterinidae (Echinodermata: Asteroidea). Memoirs of Museum Victoria. 61:1–40.

[CIT0009] O’Loughlin PM, Rowe FWE 2006 A systematic revision of the asterinid genus *Aquilonastra* O’Loughlin, 2004 (Echinodermata: Asteroidea). Memoirs of Museum Victoria. 63:257–287.

[CIT0010] Shin S 2010 Sea Star: Echinodermata: Asterozoa: Asteroidea. Invertebrate fauna of Korea, Vol. 32, No. 1 Incheon: National Institute of Biological Resources p. 1–150.

